# Supplementary material for: Predation and fragmentation portrayed in the statistical structure of prey time series
Source: BMC Ecol. 2009 May 6;9:10. doi: 10.1186/1472-6785-9-10 (PMC2689204; doi:10.1186/1472-6785-9-10)
Supplement: Additional file 2 — Voles and related classes ODDox Documentation. ODDox documentation of the agent-based model (ALMaSS) applied by Hendrichsen et al. The documentation is started by activating main.html. [file 1472-6785-9-10-S2.zip › Vole_ODDox/treatment_8h.html]

ALMaSS ODDox: treatment.h File Reference

- Main Page
- Related Pages
- Classes
- Files

# treatment.h File Reference

|  |  |
| --- | --- |
|  |  |
| Enumerations | |
| enum | FarmToDo {     start = 1, sleep\_all\_day, autumn\_plough, autumn\_harrow,     autumn\_roll, autumn\_sow, winter\_plough, deep\_ploughing,     spring\_plough, spring\_harrow, spring\_roll, spring\_sow,     fp\_npks, fp\_npk, fp\_pk, fp\_liquidNH3,     fp\_slurry, fp\_manganesesulphate, fp\_manure, fp\_greenmanure,     fp\_sludge, fa\_npk, fa\_pk, fa\_slurry,     fa\_ammoniumsulphate, fa\_manure, fa\_greenmanure, fa\_sludge,     herbicide\_treat, growth\_regulator, fungicide\_treat, insecticide\_treat,     molluscicide, row\_cultivation, strigling, flammebehandling,     hilling\_up, water, swathing, harvest,     cattle\_out, pigs\_out, cut\_to\_hay, cut\_to\_silage,     straw\_chopping, hay\_turning, hay\_bailing, stubble\_harrowing,     autumn\_or\_spring\_plough, burn\_straw\_stubble, mow, cut\_weeds,     strigling\_sow, trial\_insecticidetreat, trial\_toxiccontrol, trial\_control,     syninsecticide\_treat, cattle\_out\_low, product\_treat, glyphosate,     last\_treatment   } |

---

## Enumeration Type Documentation

enum FarmToDo

**Enumerator:**
:   |  |  |
    | --- | --- |
    | *start* |  |
    | *sleep\_all\_day* |  |
    | *autumn\_plough* |  |
    | *autumn\_harrow* |  |
    | *autumn\_roll* |  |
    | *autumn\_sow* |  |
    | *winter\_plough* |  |
    | *deep\_ploughing* |  |
    | *spring\_plough* |  |
    | *spring\_harrow* |  |
    | *spring\_roll* |  |
    | *spring\_sow* |  |
    | *fp\_npks* |  |
    | *fp\_npk* |  |
    | *fp\_pk* |  |
    | *fp\_liquidNH3* |  |
    | *fp\_slurry* |  |
    | *fp\_manganesesulphate* |  |
    | *fp\_manure* |  |
    | *fp\_greenmanure* |  |
    | *fp\_sludge* |  |
    | *fa\_npk* |  |
    | *fa\_pk* |  |
    | *fa\_slurry* |  |
    | *fa\_ammoniumsulphate* |  |
    | *fa\_manure* |  |
    | *fa\_greenmanure* |  |
    | *fa\_sludge* |  |
    | *herbicide\_treat* |  |
    | *growth\_regulator* |  |
    | *fungicide\_treat* |  |
    | *insecticide\_treat* |  |
    | *molluscicide* |  |
    | *row\_cultivation* |  |
    | *strigling* |  |
    | *flammebehandling* |  |
    | *hilling\_up* |  |
    | *water* |  |
    | *swathing* |  |
    | *harvest* |  |
    | *cattle\_out* |  |
    | *pigs\_out* |  |
    | *cut\_to\_hay* |  |
    | *cut\_to\_silage* |  |
    | *straw\_chopping* |  |
    | *hay\_turning* |  |
    | *hay\_bailing* |  |
    | *stubble\_harrowing* |  |
    | *autumn\_or\_spring\_plough* |  |
    | *burn\_straw\_stubble* |  |
    | *mow* |  |
    | *cut\_weeds* |  |
    | *strigling\_sow* |  |
    | *trial\_insecticidetreat* |  |
    | *trial\_toxiccontrol* |  |
    | *trial\_control* |  |
    | *syninsecticide\_treat* |  |
    | *cattle\_out\_low* |  |
    | *product\_treat* |  |
    | *glyphosate* |  |
    | *last\_treatment* |  |

```
00040              {
00041   start = 1,             //  1, must be first.
00042   sleep_all_day,
00043   autumn_plough,
00044   autumn_harrow,
00045   autumn_roll,
00046   autumn_sow,
00047   winter_plough,
00048   deep_ploughing,
00049   spring_plough,
00050   spring_harrow,         // 10
00051   spring_roll,
00052   spring_sow,
00053   fp_npks,
00054   fp_npk,
00055   fp_pk,
00056   fp_liquidNH3,
00057   fp_slurry,
00058   fp_manganesesulphate,
00059   fp_manure,
00060   fp_greenmanure,        // 20
00061   fp_sludge,
00062   fa_npk,
00063   fa_pk,
00064   fa_slurry,
00065   fa_ammoniumsulphate,
00066   fa_manure,
00067   fa_greenmanure,
00068   fa_sludge,
00069   herbicide_treat,  // 29
00070   growth_regulator,      // 30
00071   fungicide_treat,
00072   insecticide_treat,   // 32
00073   molluscicide,
00074   row_cultivation,
00075   strigling,      // 35
00076   flammebehandling,
00077   hilling_up,
00078   water,
00079   swathing,
00080   harvest,               // 40
00081   cattle_out,
00082   pigs_out,
00083   cut_to_hay,
00084   cut_to_silage,
00085   straw_chopping,  // 45
00086   hay_turning,
00087   hay_bailing,
00088   stubble_harrowing,
00089   autumn_or_spring_plough,
00090   burn_straw_stubble,    // 50
00091   mow,
00092   cut_weeds,
00093   strigling_sow,
00094   trial_insecticidetreat,
00095   trial_toxiccontrol,
00096   trial_control,
00097   syninsecticide_treat,
00098   cattle_out_low,
00099   product_treat,
00100   glyphosate,
00101   last_treatment // Must be last, used for size computations
00102                  // of internal arrays etc.
00103 } FarmToDo;
```

---

Generated on Thu Jan 22 14:13:45 2009 for ALMaSS ODDox by 
 1.5.6
